# Thyroid function, renal function, and depression: an association study

**DOI:** 10.3389/fpsyt.2023.1182657

**Published:** 2023-12-04

**Authors:** Hai Liang, Jin-min Wang, Xiao-qian Wei, Xiao-qin Su, Bi-xia Zhang

**Affiliations:** ^1^Department of Neurology, The Second People’s Hospital Affiliated to Fujian University of Traditional Chinese Medicine, Fuzhou, China; ^2^Department of Neurology, The Third Affiliated People’s Hospital of Fujian University of Traditional Chinese Medicine, Fuzhou, China; ^3^Department of Integrative Medicine and Psychiatry, Xiamen Xianyue Hospital, Xiamen, China; ^4^Department of Neurology, Minhou Country Hospital, Fuzhou, China

**Keywords:** thyroid function tests, renal function tests, anxiety disorders, depressive disorder, anion gap

## Abstract

**Objective:**

To investigate the correlations between thyroid function, renal function, and depression.

**Methods:**

Clinical data of 67 patients with Major depressive disorder (MDD) and 36 healthy control subjects between 2018 and 2021 were collected to compare thyroid and renal function. Thyroid and renal functions of depressed patients were then correlated with the Hamilton Depression Rating Scale (HAMD) and the Hamilton Anxiety Rating Scale (HAMA).Spearman correlation analysis was used to find the correlation between renal function, thyroid function, and depression. A logistic regression was performed to find significant predictors of depression.

**Results:**

Triiodothyronine protamine (T3), thyroxine (T4), free triiodothyronine protamine (FT3), uric acid, sodium, and anion gap were lower in the MDD group than in the control group (*p* < 0.05). Correlation analysis of thyroid function, renal function, and factor terms of HAMD in the MDD group suggested that diurnal variation, hopelessness, and depression level were positively correlated with thyrotropin (TSH) (*p* < 0.05). Cognitive disturbance, retardation, and depression level were negatively correlated with creatinine (*p* < 0.05). Diurnal variation was negatively correlated with sodium ion (*p* < 0.01); hopelessness and depression level were positively correlated with chloride ion (*p* < 0.05); diurnal variation, retardation, and depression level were negatively correlated with anion gap (*p* < 0.05). Diurnal variation (*p* < 0.01) and retardation (*p* < 0.05) were negatively correlated with osmolality. Cognitive disturbance and depression level were positively correlated with estimated glomerular filtration rate (eGFR) (*p* < 0.05). In the MDD group, correlation analysis of thyroid function, renal function, and HAMA factor terms suggested that the total HAMA score and anxiety level were positively correlated with chloride ion (*p* < 0.05); psychic anxiety, total HAMA score, and anxiety level were negatively correlated with anion gap (*p* < 0.05). Furthermore, a low level of anion gap was an independent risk factor for depression and anxiety levels (*p* < 0.05).

**Conclusion:**

Low thyroid function and reduced waste metabolized by the kidneys in patients with MDD suggest a low intake and low metabolism in depressed patients. In addition, subtle fluctuations in the anion gap in depressed patients were strongly correlated with the degree of depression and anxiety.

## 1 Introduction

Depression is a common clinical disorder characterized by significant and persistent depressed mood, with episodes of decreased interest, slowed thinking, and reduced voluntary activity, as well as somatization symptoms such as loss of appetite, weight loss, and chest tightness. From 2005 to 2015, the number of people suffering from depression increased by 18.4% worldwide and is still on the rise (1). A mental health survey showed that the lifetime prevalence of depression in China was 6.9%, and the 12-month prevalence was 3.6% (2).

Depression often coexists with other disorders, and studies have shown a reasonably strong correlation between depressive disorder and thyroid function (3, 4). Thyroid hormones are critical for brain development and mood regulation (4–6), and can act on thyroid hormone receptors in the limbic system to produce an antidepressant effect (7). Thyroid hormones can further influence anxiety and depression by modulating hippocampal levels of brain-derived neurotrophic factor (8) as well as brain-wide serotonergic signaling (9). There is evidence that triiodothyronine may accelerate the antidepressant response to antidepressants, and studies suggest that LT3 also may augment the response to antidepressants in refractory depression (10). In addition to abnormal thyroid function, chronic kidney disease (CKD) also commonly co-occurs with depression, with one study showing a five-fold increase in depression in patients with chronic kidney disease (11). A prospective cohort study with a sample size of 5,111 cases showed that for subjects experiencing depressive symptoms without renal function impairment, the prevalence of depression within 4 years was linearly related to the level of renal function (12). The interactions between depression and CKD are complex, bidirectional and multifactorial. Depression in CKD has been shown to be associated with multiple poor outcomes, including increased mortality and hospitalization rates, as well as poorer treatment compliance and quality of life (13).

The above evidence suggests that depressed patients often have thyroid or renal function abnormalities. However, there is no research yet that quantitatively analyzes the renal and thyroid functions of patients with depression, and analyzes whether there is a certain internal connection between the two in the body of patients with depression. Thyroid hormone levels and renal function levels can indirectly reflect human metabolism and excretion. Based on previous research observations, we speculate that depressed patients often exhibit physiological states of low intake, low metabolism, and low excretion, possibly accompanied by low levels of thyroid and renal function.The aim is to explore the metabolic changes in the kidney and thyroid function in patients with major depressive disorder, and to search for possible pathogenic factors and disease markers in patients with depression.

## 2 Materials and methods

### 2.1 Subjects.

Healthy subjects were invited to participate in the study if they had undergone a physical examination at our hospital between January 2019 and February 2021. Patients diagnosed with Major depressive disorder (MDD) and receiving antidepressant treatment at our hospital for the first time between January 2019 and February 2021 who met the inclusion and exclusion criteria were also enrolled. MDD was diagnosed according to the diagnostic criteria for depressive disorder in the Diagnostic and Statistical Manual of Mental Disorders-5 (DSM-5) (14).

### 2.2 Inclusion and exclusion criteria

Inclusion criteria for the MDD group were as follows: (1) aged 18–65 years, (2) two psychiatrists provide independent diagnosis for MDD based on structured clinical interviews of the DSM-5, (3) aware of and willing to complete the Hamilton Depression Rating Scale (HAMD) and Hamilton Anxiety Scale (HAMA) survey and assessment, (4) no previous diagnosis of depression and not previously treated with antidepressant therapy, (5) not taking drugs affecting renal or thyroid function within three months of enrollment, (6) voluntarily participated in the clinical trial and provided signed informed consent.

The inclusion criteria for the control group were as follows: (1) aged 18–65 years, (2) capable of communicating normally and without harsh language or hearing impairment, (3) HAMD score < 8, (4) formal neuropsychological testing is conducted by trained professionals and includes evaluations of depression and mania dimensions to rule out patients with mental disorders.

The exclusion criteria were as follows: (1) pregnant or lactating, (2) addicted to or reliant on alcohol or drugs, (3) severe heart, brain, or kidney diseases; (4) awareness disorders, aphasia, or agnosia preventing them from communicating; (5) history of hypertension, hyperlipidemia, myocardial infarction, coronary atherosclerotic heart disease, or cerebral infarction; (6) currently suffering from hematological diseases; (7) infectious diseases, abscesses, pneumonia, or chronic obstructive pulmonary disease; (8) received hormonal drugs or contraceptives within the previous six months; (9) kidney damaging drugs such as antibiotics, NSAIDs, anti-tumors, contrast agents, immunosuppressants have been used in the last three months. (10) drugs that affect thyroid hormone levels such as iodine, hormones, interferons, NSAIDs, antitumor agents, contrast agents, immunosuppressants, etc., have been used in the last three months.

### 2.3 Grouping

From January 2019 to February 2021, a total of 98 healthy subjects were evaluated in our hospital, 62 of whom were excluded (56 refused to participate, 2 had incomplete data, 1 had a family history of mental illness, 3 were suspected to be mildly depressed and had abnormal HAMD and HAMA total scores). The remaining 36 healthy subjects were set as the control group. During the same time period a total of 179 MDD patients were evaluated in our hospital, 112 of whom were excluded according to the criteria listed above (55 refused to participate, 12 had incomplete data, 18 had a family history of mental illness, 27 had schizophrenia or other psychotic disorders). The 67 eligible MDD patients were set as the study group.

The present study was performed according to the principles of the Helsinki Declaration of the World Medical Association. It was approved by the Ethics Committee of The Second People’s Hospital Affiliated with Fujian University of Traditional Chinese Medicine (ethical approval: 2015-KL005-01). All participants provided signed informed consent.

### 2.4 Data collection

The scales used in this study were translated from the HAMD and HAMA compiled by Hamilton. The scales have been evaluated for application in China. His reliability coefficient is 0.93, and the reliability coefficient of each individual symptom score is 0.83–1.00, with value of ps less than 0.01. Its validity coefficient is 0.36 (*p* < 0.05). The day before blood sample collection, each patient completed the HAMD (less than 8 points indicate no depression; 8–20 points indicate mild depression; 21–35 points indicate moderate depression; greater than 35 points indicate severe depression) and HAMA (less than 6 points indicate no anxiety; 6–14 points indicate mild anxiety; 15–21 points indicate moderate anxiety; greater than 21 points indicate severe anxiety) assessments as instructed by a full-time staff member. Demographic data (sex, age, height, weight, education, smoking, drinking, and heart rate) and assessment scores (HAMD and HAMA) were recorded in the case file. On the following day, between 07:30 and 08:30 AM, elbow vein blood was collected under fasting status.

Triiodothyronine protamine (T3) (normal: 0.99–2.72 nmol/L), thyroxine (T4) (normal: 78.38–157.40 nmol/L), protamine (FT3) (normal: 3.28–7.42 pmol/L), FT4, free thyroxine (normal: 7.5–21.1 pmol/L), and thyrotropin (TSH) (normal: 0.34–5.6 mIU/L), thyroglobulin antibodies (TGAb) (normal: 0–4 IU/mL), and thyroid peroxidase antibodies (TPOAb) (normal: 0–9 IU/mL) were measured by means of chemiluminescence with the Beckman-Coulter Unicel DXi800 immunoanalyzer (Beckman Coulter Inc., Brea, CA, USA). Using the Abbott Architect c16000 automatic biochemical analysis system (Abbott Laboratories, Chicago, IL, USA), urea (normal: 3–7.8 mmol/L) and creatinine (normal: 40–120 umol/L) were measured by enzymatic method; uric acid (umol/L), calcium ion (mmol/L), magnesium ion (mmol/L), and phosphorus ion (mmol/L) were measured by colorimetry method; and sodium (mmol/L), potassium (mmol/L), and chloride (mmol/L) ions were measured by electrode method. The estimated glomerular filtration rate (eGFR) was calculated using the simplified diet modification in renal disease formula described by the National Kidney Foundation as follows: eGFR (mL/min/1.73 m^2^) = 175 × (Scr)^-1.154^ × (Age)^-0.203^ × (0.742 if female) × (1.212 if African American) (15).

### 2.5 Statistical analysis

Continuous and categorical variables were summarized as mean (standard deviation), median (inter-quartile range, 1st quartile–3rd quartile), and frequency (percentage). Continuous variables with a normal distribution were compared using the t-test; otherwise, the Wilcoxon rank-sum test was used, confirmed by the Shapiro–Wilk test. Categorical variables were compared using the Chi-square test or Fisher’s exact test. Thyroid function, renal function, and electrolytes were analyzed by Spearman correlation with HAMD score, and the Spearman correlation coefficient (r) and value of p were obtained. Parameters that were found to be statistically significant by Spearman correlation analysis were included in the multivariate ordered logistic regression analysis to obtain the regression coefficient, odds ratio (OR), and value of p. Thyroid function, renal function, and electrolytes were analyzed by Spearman correlation with HAMA score, and the Spearman correlation coefficient (r) and value of p were obtained. Parameters that were found to be statistically significant by Spearman correlation analysis were included in the multiple linear regression analysis to obtain the regression coefficient and value of *p*. The significance level was set at *p* < 0.05. All statistical analyses were performed using IBM SPSS software version 26.0.

## 3 Results

### 3.1 Demographic characteristics

There was no significant difference between control and MDD groups in terms of sex, age, height, weight, education, smoking, drinking, and heart rate (*p* > 0.05) (see Table 1).

**Table 1:** Demographic characteristics of the participants.

		MDD group (*n* = 67)	Control group (*n* = 36)	t/z/**χ**^2^	*p* value
Gender	Male	20(29.9%)	16(44.4%)	2.194	0.139
	Female	47(70.1%)	20(55.6%)		
Age (years)		39.00 (31.50, 52.00)	50.00 (38.00, 55.00)	−1.845	0.065
Height (cm)		163.26 ± 9.08	164.87 ± 8.94	0.809	0.421
Weight (kg)		58.00 (52.00, 66.50)	61.50 (55.00, 72.40)	−1.218	0.223
Marriage	Unmarried	13 (65.0%)	7 (35.0%)	0.000	0.996
	Married	54 (65.1%)	29 (34.9%)		
Education level (years)		12.00 (7.00, 15.00)	12.00 (9.00, 12.00)	−0.260	0.795
Smoking	Yes	3(4.5%)	3(8.3%)	0.126	0.722
	No	64(95.5%)	33(91.7%)		
Drinking	Yes	5(7.5%)	4(11.1%)	0.067	0.795
	No	62(92.5%)	32(88.9%)		
Heart rate (times/min)		78.00 (70.50, 90.00)	79.50 (72.00, 89.00)	−0.391	0.696

*p < 0.05.

### 3.2 Comparison of thyroid function, renal function, and electrolyte levels

Serum levels of T3, T4, and FT3 in the MDD group were significantly lower than in the control group (*p* < 0.05). There was no significant difference in serum FT4, TSH, TGAb, or TPOAb between the two groups (*p* > 0.05).Serum uric acid, sodium ion, and osmolality were significantly lower in the MDD group than in the control group (all p < 0.05). There was no significant difference in serum urea, creatinine, potassium ion, chloride ion, calcium ion, magnesium ion, phosphorus ion, anion gap, or eGFR between the two groups (all *p* > 0.05) (Table 2).

**Table 2:** Comparison of thyroid function, renal function, and electrolytes between the two groups.

	MDD group (*n* = 67)	Control group (*n* = 36)	t/z	value of *p*
T3 (nmol/L)	1.51 (1.36, 1.73)	1.74 (1.45, 2.01)	−2.141	0.032*
T4 (nmol/L)	109.30 (99.00, 125.02)	125.25 (105.46, 134.78)	−2.172	0.030*
TSH (mIU/L)	1.59 (0.98, 2.27)	1.48 (0.73, 2.55)	−0.661	0.509
FT3 (pmol/L)	5.12 (4.54, 5.61)	5.50 (4.95, 5.98)	−2.283	0.022*
FT4 (pmol/L)	12.08 (10.89, 13.51)	11.14 (10.51, 13.81)	−0.878	0.380
TGAb (IU/mL)	0.10 (0.10, 2.25)	0.10 (0.10, 0.20)	−1.812	0.070
TPOAb (IU/mL)	1.20 (0.50, 12.40)	1.25 (0.40, 5.35)	−0.481	0.630
Urea (mmol/L)	4.00 (3.50, 4.95)	4.45 (3.80, 5.40)	−1.962	0.050
Creatinine (umol/L)	60.20 (55.65, 73.10)	61.50 (53.50, 74.00)	−0.259	0.795
Uric acid (umol/L)	281.00 (235.97, 342.84)	339.28 (281.88, 390.86)	−2.777	0.005**
Sodium ion (mmol/L)	139.80 (138.30, 141.15)	140.95 (139.30, 142.10)	−2.152	0.031*
Potassium ion (mmol/L)	4.09 ± 0.32	4.19 ± 0.42	1.277	0.204
Chloride ion (mmol/L)	105.25 (102.85, 105.80)	105.04 (103.20, 106.90)	−0.426	0.670
Calcium ion (mmol/L)	2.33 ± 0.10	2.34 ± 0.12	0.415	0.679
Magnesium ion (mmol/L)	0.93 (0.89, 0.97)	0.92 (0.86, 0.98)	−0.367	0.714
Phosphorus ion (mmol/L)	1.19 ± 0.18	1.20 ± 0.13	0.340	0.734
Anion gap (mmol/L)	10.70 (9.50, 11.55)	10.60 (9.85, 12.10)	−0.287	0.774
Osmolality (mosm/L)	290.22 (287.56, 292.22)	292.38 (288.89, 294.98)	−2.283	0.022*
eGFR (ml/min/1.73m^3^)	102.61 (91.62, 111.72)	99.18 (91.28, 120.33)	−0.588	0.557

T3, triiodothyronine; T4, thyroxine; TSH, thyrotropin; FT3, free triiodothyronine; FT4, free thyroxine; TGAb, thyroglobulin antibodies; TPOAb, thyroid peroxidase antibodies; eGFR, estimated glomerular filtration rate; *p < 0.05; **p < 0.01.

### 3.3 Exploratory analysis of FT4

There were significant group differences in the distributions of T3, T4, and FT3, but not FT4. To further explore the distribution of FT4, a regression model was established with FT4 as the dependent variable. However, since FT4 did not follow a normal distribution, the inverse of FT4 was used in the regression. Potential influencing factors (depression, gender, age, years of education, marital status, height, weight, heart rate, systolic blood pressure, diastolic blood pressure, total depression score, total anxiety score) were used as independent variables to establish a multiple stepwise linear regression model. Of all the independent variables investigated, only heart rate and gender appeared to affect FT4 distribution (Table 3), and serum FT4 levels were positively correlated with heart rate.

**Table 3:** Multiple stepwise linear regression analysis.

Variables	b	S_b_	β	t	*value of p*	Collinearity statistics	
						Tolerance	VIF
Heart rate (beats/min)	−0.000	−0.000	−0.329	−3.278	0.002*	0.999	1.001
Gender	0.009	0.004	0.251	2.498	0.015*	0.999	1.001

### 3.4 Correlation of thyroid and renal functions with HAMD scores in MDD patients

#### 3.4.1 Thyroid and renal functions correlate with HAMD factor scores

**Figure 1:**
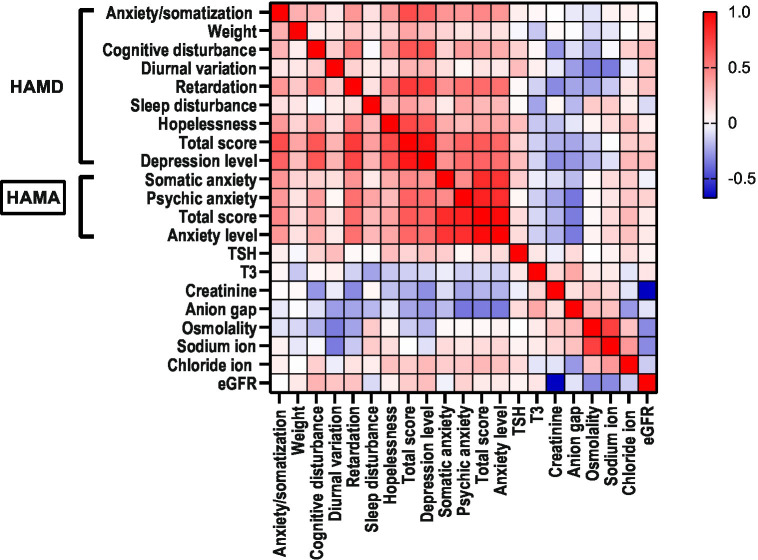
Heatmap of correlation analysis.

In the MDD group, diurnal variation (R = 0.258, *p* < 0.05), hopelessness (R = 0.255, *p* = 0.038), and depression level (R = 0.252, *p* < 0.05) were positively correlated with serum levels of TSH, and sleep disturbance (R = −0.245, *p* < 0.05) was negatively correlated with serum T3. cognitive disturbance (R = −0.277, *p* < 0.05), retardation (R = −0.309, *p* < 0.05), and depression level (R = −0.300, *p* < 0.05) were negatively correlated with creatinine; diurnal variation (R = −0.349, *p* < 0.01) was negatively correlated with sodium ion; hopelessness (R = 0.247, *p* < 0.05) and depression level (R = 0.272, *p* < 0.05) were positively correlated with chloride ion; diurnal variation (R = −0.262 *p* < 0.05), retardation (R = −0.242, *p* < 0.05), and depression level (R = −0.280, *p* < 0.05) were negatively correlated with anion gap; diurnal variation (R = −0.346, *p* < 0.01) was negatively correlated with osmolality; retardation (R = −0.243, *p* < 0.05) was negatively correlated with osmolality; cognitive disturbance (R = 0.295, *p* < 0.05) and depression level (R = 0.253, *p* < 0.05) were positively correlated with eGFR (Table 4; Figure 1).

**Table 4:** Correlation analysis of thyroid and renal function and HAMD factor scores in MDD patients.

		Anxiety/somatization	Weight	Cognitive disturbance	Diurnal variation	Retardation	Sleep disturbance	Hopelessness	Total score	Depression level
T3 (nmol/L)	r	0.034	−0.148	0.032	0.065	−0.126	−0.245*	−0.148	−0.128	−0.117
	*P*	0.788	0.231	0.800	0.603	0.309	**0.046**	0.231	0.303	0.344
T4 (nmol/L)	r	0.175	0.026	0.036	−0.205	−0.005	−0.007	−0.096	0.025	−0.005
	*P*	0.157	0.837	0.774	0.096	0.971	0.953	0.440	0.843	0.970
TSH (mIU/L)	r	0.063	−0.021	0.173	0.258*	0.024	0.016	0.255*	0.170	0.252*
	*P*	0.612	0.865	0.161	**0.035**	0.848	0.897	**0.038**	0.170	**0.040**
FT3 (pmol/L)	r	0.024	−0.095	0.009	0.191	−0.153	−0.113	−0.127	−0.078	−0.027
	*P*	0.848	0.443	0.943	0.121	0.216	0.362	0.306	0.531	0.828
FT4 (pmol/L)	r	0.095	0.072	0.103	−0.168	−0.104	0.043	−0.167	0.028	0.047
	*P*	0.443	0.560	0.409	0.173	0.401	0.731	0.176	0.822	0.707
TGAb (IU/mL)	r	−0.032	−0.046	−0.047	−0.095	−0.075	−0.065	0.177	−0.054	0.018
	*P*	0.796	0.711	0.705	0.444	0.544	0.601	0.151	0.667	0.882
TPOAb (IU/mL)	r	0.112	−0.064	−0.014	−0.094	0.059	−0.061	0.137	0.035	0.028
	*P*	0.367	0.607	0.909	0.449	0.636	0.626	0.268	0.780	0.821
Urea (mmol/L)	r	−0.031	−0.066	−0.043	−0.110	−0.097	0.120	−0.021	−0.024	−0.013
	*P*	0.803	0.596	0.727	0.375	0.433	0.334	0.865	0.847	0.919
Creatinine (umol/L)	r	0.005	0.023	−0.277^*^	−0.057	−0.309^*^	0.033	−0.166	−0.212	−0.300^*^
	*P*	0.969	0.854	**0.023**	0.646	**0.011**	0.790	0.179	0.085	**0.014**
Uric acid (umol/L)	r	0.011	0.002	−0.077	0.141	−0.145	0.046	−0.156	−0.066	−0.113
	*P*	0.930	0.986	0.535	0.255	0.241	0.713	0.207	0.595	0.363
Sodium ion (mmol/L)	r	0.050	−0.065	−0.020	−0.349^**^	−0.148	0.204	0.133	−0.005	−0.084
	*P*	0.686	0.603	0.872	**0.004**	0.234	0.098	0.283	0.971	0.499
Potassium ion (mmol/L)	r	−0.064	−0.099	0.154	−0.157	−0.026	−0.030	−0.053	−0.019	−0.005
	*P*	0.609	0.426	0.214	0.204	0.837	0.807	0.670	0.882	0.969
Chloride ion (mmol/L)	r	0.060	−0.004	0.190	−0.050	0.106	0.074	0.247^*^	0.199	0.272^*^
	*P*	0.627	0.975	0.123	0.690	0.391	0.551	**0.044**	0.106	**0.026**
Calcium ion (mmol/L)	r	−0.015	0.151	0.051	−0.042	−0.200	−0.030	0.002	−0.030	−0.115
	*P*	0.905	0.223	0.684	0.736	0.105	0.809	0.985	0.810	0.353
Magnesium ion (mmol/L)	r	−0.002	0.116	0.213	−0.024	0.101	−0.114	0.112	0.092	0.021
	*P*	0.984	0.351	0.083	0.850	0.415	0.359	0.368	0.458	0.866
Phosphorus ion (mmol/L)	r	0.085	0.072	0.017	0.078	−0.196	−0.042	0.127	0.031	−0.027
	*P*	0.496	0.562	0.894	0.529	0.113	0.734	0.305	0.803	0.828
Anion gap (mmol/L)	r	−0.060	−0.008	−0.087	−0.262^*^	−0.242^*^	−0.189	−0.067	−0.237	−0.280^*^
	*P*	0.628	0.947	0.485	**0.032**	**0.048**	0.126	0.591	0.054	**0.022**
Osmolality (mosm/L)	r	−0.077	−0.104	−0.207	−0.346^**^	−0.243^*^	0.204	0.040	−0.137	−0.193
	*P*	0.538	0.402	0.093	**0.004**	**0.048**	0.098	0.747	0.269	0.118
eGFR (ml/min/1.73m^3^)	r	−0.004	0.088	0.295^*^	0.229	0.235	−0.099	0.062	0.198	0.253^*^
	*P*	0.977	0.480	**0.016**	0.063	0.055	0.424	0.619	0.109	**0.039**

T3, triiodothyronine; T4, thyroxine; TSH, Thyrotropin; FT3, free triiodothyronine; FT4, free thyroxine; TGAb, thyroglobulin antibodies; TPOAb, thyroid peroxidase antibodies; eGFR, estimated glomerular filtration rate; *p < 0.05.

#### 3.4.2 Multivariate ordered logistic regression analysis for depression level

Five variables that were statistically significant in the above correlation analyses (TSH, creatinine, chloride, anion gap, and eGFR) were selected as independent variables and the level of depression was used as the dependent variable for further multivariate logistic regression analysis. The analysis showed that a low level of anion gap was an independent risk factor for depression level; its OR value was greater than 1 and its value of *p* was less than 0.05 (Table 5).

**Table 5:** Multivariate ordered logistic regression analysis for depression level.

Variables	b	S_b_	Waldχ^2^	*value of p*	OR	95% CI	
						Lower	Upper
TSH	0.284	0.172	2.707	0.100	1.328	0.947	1.863
Creatinine	−0.018	0.027	0.440	0.507	0.982	0.931	1.036
Chloride ion	0.143	0.133	1.143	0.285	1.154	0.888	1.498
Anion gap	−0.348	0.153	5.145	0.023*	0.706	0.523	0.954
eGFR	0.016	0.018	0.760	0.383	1.016	0.98	1.052

b, partial regression coefficient; Sb, standard error; OR, odds ratio; CI, confidence interval; TSH, Thyrotropin; eGFR, estimated glomerular filtration rate; *p < 0.05.

### 3.5 Correlation of thyroid and renal functions with HAMA scores in MDD patients

#### 3.5.1 Thyroid functions and renal functions correlate with HAMA factor scores

In the MDD group, there were no significant correlations observed between any thyroid functions and HAMA factors. The total HAMA score (R = 0.276, *p* < 0.05) and anxiety level (R = 0.246, *p* < 0.05) were positively correlated with chloride ion. Psychic anxiety (R = −0.362, *p* < 0.01), total HAMA score (R = −0.346, *p* < 0.01), and anxiety level (R = −0.350, *p* < 0.01) were negatively correlated with anion gap (Table 6; Figure 1).

**Table 6:** Correlation analysis of thyroid and renal function and HAMA factor score in MDD patients.

		Somatic anxiety	Psychic anxiety	Total score	Anxiety level
T3 (nmol/l)	r	−0.052	−0.128	−0.109	−0.130
	*P*	0.674	0.301	0.380	0.296
T4 (nmol/l)	r	0.100	0.149	0.119	0.129
	*P*	0.419	0.230	0.339	0.298
TSH (mIU/l)	r	0.192	0.033	0.137	0.134
	*P*	0.120	0.793	0.269	0.281
FT3 (pmol/l)	r	−0.027	−0.060	−0.064	−0.079
	*P*	0.830	0.627	0.609	0.528
FT4 (pmol/l)	r	−0.057	0.043	0.005	0.002
	*P*	0.647	0.729	0.970	0.988
TGAb (IU/ml)	r	0.075	−0.090	−0.013	−0.026
	*P*	0.546	0.470	0.914	0.836
TPOAb (IU/ml)	r	0.024	−0.024	−0.002	−0.056
	*P*	0.849	0.846	0.984	0.653
Urea (mmol/L)	r	−0.013	0.071	0.026	0.054
	*P*	0.915	0.569	0.832	0.662
Creatinine (umol/L)	r	−0.093	−0.240	−0.197	−0.204
	*P*	0.455	0.050	0.11	0.098
Uric acid (umol/L)	r	0.063	−0.039	−0.014	0.007
	*P*	0.611	0.757	0.907	0.956
Sodium ion (mmol/L)	r	0.139	0.096	0.14	0.161
	*P*	0.261	0.438	0.258	0.193
Potassium ion (mmol/L)	r	−0.084	0.112	0.028	0.004
	*P*	0.499	0.365	0.822	0.972
Chloride ion (mmol/L)	r	0.196	0.217	0.276*	0.246*
	*P*	0.113	0.077	**0.024**	**0.044**
Calcium ion (mmol/L)	r	0.017	−0.198	−0.135	−0.139
	*P*	0.890	0.108	0.275	0.263
Magnesium ion (mmol/L)	r	−0.157	−0.139	−0.157	−0.129
	*P*	0.204	0.262	0.206	0.298
Phosphorus ion (mmol/L)	r	0.240	−0.012	0.129	0.139
	*P*	0.050	0.922	0.299	0.262
Anion gap (mmol/L)	r	−0.182	−0.362**	−0.346**	−0.350**
	*P*	0.141	**0.003**	**0.004**	**0.004**
Osmolality (mosm/L)	r	0.024	0.027	0.026	0.050
	*P*	0.847	0.827	0.837	0.689
eGFR (ml/min/1.73m^3^)	r	−0.038	0.176	0.079	0.087
	*P*	0.762	0.154	0.528	0.483

T3, triiodothyronine; T4, thyroxine; TSH, Thyrotropin; FT3, free triiodothyronine; FT4, free thyroxine; TGAb, thyroglobulin antibodies; TPOAb, thyroid peroxidase antibodies; eGFR, estimated glomerular filtration rate; *p < 0.05; **p < 0.01.

#### 3.5.2 Multiple linear regression analysis for total HAMA score

Two variables that were statistically significant in the above correlation analysis (chloride ion and anion gap) were selected as independent variables (*p* < 0.05), and the total HAMA score was used as the dependent variable for further multivariate logistic regression analysis. The analysis showed that anion gap was an independent factor affecting the total HAMA score; the lower the anion gap, the higher the total HAMA score. On the other hand, chloride ion had no direct impact on the total HAMA score (Table 7).

**Table 7:** Multiple linear regression analysis for the total HAMA score.

Variables	b	S_b_	β	t	*p*-value	Collinearity statistics	
						Tolerance	VIF
Anion gap	−1.22	0.520	−0.291	−2.344	0.022*	0.900	1.111
Chloride ion	0.392	0.501	0.097	0.783	0.437	0.900	1.111

*R*^2^ = 0.112; Sb, standard error; β, standardized coefficients; VIF, variance inflation factor; *p < 0.05.

## 4 Discussion

This study showed that serum T3, T4, and FT3 levels were significantly lower in depressed patients than in normal controls, suggesting that depressed patients have lower levels of thyroid function. The thyroid gland is the largest endocrine gland in the human body and secretes thyroid hormone, which regulates the body’s growth, development, metabolism, and other functional activities. It is generally accepted that depressed patients have abnormalities in the hypothalamic–pituitary-thyroid axis, which manifest as significant thyroid dysfunction (16, 17). The American Association of Endocrinologists also stated that depression must be considered in all patients diagnosed with subclinical or clinical hypothyroidism (18). Therefore, there is a close relationship between low thyroid function and depression. In addition, hypothyroidism is one of the leading causes of refractory depression, and thyroid replacement therapy for underlying hypothyroidism can significantly improve mood disorders such as depression (19).

Unlike the results of previous studies, the present study showed no statistical difference in FT4 and TSH between MDD and control groups. In the correlation analysis, serum TSH was positively correlated with diurnal variation, hopelessness, and depression level. This aligns with previous studies (20–23) that demonstrated a positive relationship between circulating TSH levels and total HAMD score and duration of illness, suggesting that TSH may be a useful predictor of depression severity and duration. As for FT4, this study analyzed many potential factors and concluded that the most influential were heart rate and gender. However, the b-value for heart rate was low, and clinical evidence for an effect of heart rate of FT4 is weak. In contrast, evidence for an effect of gender is much stronger. FT4 in serum is known to be higher in men than in women (24), and our relatively small sample size may have exacerbated this natural variation. Together these results failed to demonstrate a difference in FT4 between depressed and non-depressed populations. However, there was a difference across genders, with higher levels of serum FT4 in males than females.

Renal function refers to the ability of the kidneys to excrete metabolic wastes from the body and maintain stable levels of electrolytes and acidity. Standard clinical tests for renal function include blood creatinine, urea, and uric acid. Data from a cross-sectional study of a Chinese population showed that both the degree of depression and depressive symptoms were significantly correlated with the level of renal function and remained significant even after excluding confounding factors such as chronic kidney disease, hypertension, and diabetes, suggesting that minor changes in early renal function may have some correlation with depression (25). In recent years, the concept of a brain-kidney axis has been proposed based on the vascular and hemodynamic similarities between kidney and brain. It is thought that microvascular damage in the kidney reflects the situation in the brain and there may even be crosstalk between the two organs (26). These studies provide a theoretical basis to explain the interaction between somatic symptom disorders and depression.

There are two types of blood creatinine: exogenous and endogenous. Exogenous creatinine comes from the absorption and metabolism of meat products; endogenous creatinine is a product of muscle metabolism. Creatinine is formed slowly in muscle through an irreversible non-enzymatic dehydration reaction and then released into the blood. Depressed patients fatigue easily and generally engage in less active exercise, so muscle tissue metabolism is slowed and the body produces less endogenous creatinine. Studies have shown a negative relationship between depression and blood creatinine, with lower blood creatinine values associated with higher depression scores (27). A prospective study of the general population, with normal renal function, showed that the presence of depressive symptoms was correlated with blood creatinine, urea, and serum cystatin C levels over 4 years (12). The present results are consistent with these other findings. Although no significant differences were observed in the distribution of creatinine between MDD and control groups, within the MDD group blood creatinine levels were negatively correlated with HAMD total score as well as cognitive disturbance and retardation factor scores. Thus, the lower the blood creatinine level of depressed patients, the more severe their depression appears to be, particularly with regard to cognitive impairment and delayed thinking and behavior.

Like creatinine, uric acid can also be derived exogenously or endogenously. Uric acid is the end product of purine metabolism, and is endogenously produced by degrading the nucleic acid bases adenine and guanine of normal or dying cells; its exogenous source is the metabolism of ingested animal proteins (28, 29). Uric acid is mainly excreted through the kidneys, and elevated serum levels of uric acid can cause gout, dyslipidemia, cardiovascular disease, hypertension, and other diseases (30–32). In recent years it has been noted as a potential marker of adenosine transmission dysfunction and may therefore be useful in diagnosing affective disorders (33–35). The present study found that circulating uric acid levels in depressed patients were significantly lower than in normal controls. It has been shown that patients with monophasic depressive disorder have lower blood uric acid levels (36, 37); in contrast, patients with bipolar disorder had higher uric acid levels, especially during manic episodes (38–40) in which patients exhibit altered affective states and behavior, including impulsivity and aggression (34). Therefore, uric acid shows promise as a sensitive biological indicator of negative and positive symptoms in bipolar disorder, and may also be useful for determining prognosis in major depressive disorder (39, 41). Since the present study specifically excluded patients with suspected bipolar disorder, our results can only support the association of uric acid levels with MDD.

Anion gap is a marker of blood acidity that is calculated by subtracting the major serum anions (Cl^−^ and HCO_3_^−^) from the major serum cations (Na^+^ and K^+^). It is an artificially conceived value that only reflects the difference between anions and cations routinely measured in the laboratory. According to the principle of electroneutrality, the sum of measured anions in serum is equal to the sum of cations. Therefore, anion gap reflects the difference between unmeasured anions and unmeasured cations in plasma. The results of this study suggest that the degree of depression is negatively related to anion gap, positively related to chloride ions, and not related to sodium and potassium ions. According to the anion gap formula, the negative correlation between anion gap and depression may be due to the positive correlation between chloride ion and depression. Regression analysis of anion gap with HAMA and HAMD showed that anion gap was correlated with anxiety and depression, suggesting that higher serum levels of chloride were associated with higher levels of anxiety and depression. Possible unmeasured anions that were accounted for in the anion gap include low-level anions and negatively charged proteins in serum. One study (42) of T4 binding demonstrated that several different anions have the ability to displace thyroxine from binding sites on human serum albumin, and serum concentrations of chloride ions and protein can affect the concentration of T4. High chloride ion concentration can therefore inhibit the binding of T4 so that FT4 does not decrease, as observed in the present study in the serum of patients with depression. A study (43) found that after adjusting the effect of albumin and chloride concentrations on thyroid hormone binding in plasma, the correlation between thyroid hormone index and depression became insignificant. This suggests that concentrations of chloride ion and protein in serum may underlie abnormal thyroid hormone in patients with depression. Therefore, it is believed that there may be a relationship between serum chloride level and thyroid function, as well as a positive correlation with the degree of depression. However, the mechanism underlying the relationship between chloride level and depression severity remains unclear.

Based on the results of this study, we found that as the severity of depression increases in patients with major depression, the body exhibits a state of low energy consumption and low metabolism. Based on the correlation heatmap analysis, we speculate that when the human body is in a high-level state of depression, the metabolic level decreases, which is manifested by a decrease in creatinine production, while compensatoryly increasing TSH to counter the low energy consumption state caused by depression. Overall, patients with major depression exhibit a decrease in thyroid function and renal function-related indicators at a low level. In addition, the internal environment can also be affected to some extent, and the subtle fluctuations in the anion gap in depressed patients are closely related to the degree of depression and anxiety.

Most previous studies have recognized that depression is related to thyroid dysfunction or renal dysfunction. However, few studies have simultaneously focused on the relationship and changes between thyroid function and renal function in patients with depression. This study found that there may be a certain correlation between renal and thyroid dysfunction levels in patients with depression, and it is related to the metabolic levels in the body of patients with depression. However, the current research results are not sufficient to support the elucidation of the connection mechanism between thyroid function and renal function. It is also unclear whether it has a certain correlation with the onset and progression of depression. In addition, due to the fact that this study is not a cohort study, the changes in thyroid and renal function of enrolled patients after antidepressant treatment cannot be determined, and there is a lack of follow-up research. Therefore, whether there are internal biological signaling pathways in thyroid and renal functions, how thyroid and renal function change during the treatment process of depression patients, and whether there is a relationship between the distribution characteristics of anions in the internal environment of depression patients and the pathogenesis of depression will be the next research direction.

## Data availability statement

The original contributions presented in the study are included in the article/Supplementary material, further inquiries can be directed to the corresponding author.

## Ethics statement

The studies involving humans were approved by the Ethics Committee of The Second People’s Hospital Affiliated with Fujian University of Traditional Chinese Medicine. The studies were conducted in accordance with the local legislation and institutional requirements. The participants provided their written informed consent to participate in this study.

## Author contributions

HL and J-mW was the principal investigator, oversaw data collection, conceived the study, and contributed to writing—original draft, review and editing. X-qW contributed to the scoring/management of the data and writing original draft, review, and editing. X-qS conducted the data analysis and contributed to writing original draft, review and editing. All authors contributed to the article and approved the submitted version.
