# Supplementary material for: Thyroid function, renal function, and depression: an association study
Source: Front Psychiatry. 2023 Dec 4;14:1182657. doi: 10.3389/fpsyt.2023.1182657 (PMC10765600; doi:10.3389/fpsyt.2023.1182657)
Supplement: Supplementary file 1 [file Data_Sheet_1.docx]

Hamilton depression scale (HAMD)

Hamilton depression scale (HAMD), compiled by Hamilton in 1960, is the most commonly used scale in clinical evaluation of depression, and is often used as a tool for parallel validity test of other depression scales. It is mainly applicable to people with depressive symptoms such as depression.

[Evaluation criteria]

Most HAMD projects adopt a five-level scoring method of 0-4 points: 0-none; 1 point - mild; 2 points - moderate; 3 points - severe; 4 points - very heavy. The 3-level scoring method of 0-2 points for a few items: 0 - none; 1 point - light to moderate; 2 points - severe.

1 Depressed mood 0–4

2 Low self-esteem, guilt 0–4

3 Suicidal thoughts 0–4

4 Insomnia: initial 0–2

5 Insomnia: middle 0–2

6 Insomnia: late 0–2

7 Work and interests 0–4

8 Psychomotor retardation 0–4

9 Psychomotor agitation 0–4

10 Anxiety, psychic 0–4

11 Anxiety, somatic 0–4

12 Gastrointestinal symptoms (appetite) 0–2

13 Somatic symptoms, general 0–2

14 Sexual disturbances 0–2

15 Hypochondriasis (somatisation) 0–4

16 Insight 0–2

17 Weight loss 0–2

18 Diurnal variation 0–2

19 Depersonalization and derealisation 0–4

20 Paranoid symptoms 0–4

21 Obsessional and compulsive symptoms 0–2

22 Helplessness 0–4

23 Hopelessness 0–4

24 Worthlessness 0–4

[Result analysis]

1. Total score

The sum of the individual parts. Reflect the severity of the disease. According to the cut-off value of Davis JM, the total score exceeds 35 points, which may be severe depression; More than 20 points may be mild to moderate depression; If the score is less than 8, there is no depressive symptom. The 17 versions (i.e. 1-17 items) are 24 points, 17 points and 7 points. China's 14 units cooperated to study 115 inpatients with depression, that is, a group of patients with severe depression. The results showed that the total score of 17 versions of HAMD was 28.45 ± 7.16, which can be used for analogy and reference

2. Factor score

The sum of the scores of each component factor/the number of items that make up the factor. HAMD can be summarized into 7 types of factors:

(1) Anxiety/somatization: composed of 6 items including 10, 11, 12, 13, 15 and 17

(2) Weight: item 16

(3) Cognitive disturbance: composed of 6 items including 2, 3, 9, 19, 20 and 21

(4) Diurnal variation: item 18

(5) Retardation: composed of items 1, 7, 8 and 14

(6) Sleep disturbance: composed of items 4, 5, 6, etc

(7) Hopelessness: composed of items 22, 23 and 24

Hamilton anxiety scale (HAMA)

Hamilton anxiety scale (HAMA) was prepared by Hamilton in 1959. It is one of the commonly used clinical scales in psychiatry. It is mainly used to assess the severity of anxiety in neurosis and other patients.

[Evaluation criteria]

All items of HAMA adopt the 5-level scoring method of 0-4 points:

0 point - no symptoms;

1 point - light;

2 points - medium (positive symptoms, but not affecting life and activities);

3 points - severe (severe symptoms, need to be treated or have affected life activities);

4 points - extremely serious (symptoms are extremely serious, seriously affecting life).

1. ANXIOUS MOOD

2. TENSION

3. FEARS

4. INSOMNIA

5. INTELLECTUAL

6. DEPRESSED MOOD

7. SOMATIC COMPLAINTS: MUSCULAR

8. SOMATIC COMPLAINTS: SENSORY

9. CARDIOVASCULAR SYMPTOMS

10. RESPIRATORY SYMPTOMS

11. GASTROINTESTINAL SYMPTOMS

12. GENITOURINARY SYMPTOMS

13. AUTONOMIC SYMPTOMS

14. BEHAVIOR AT INTERVIEW

[Result analysis]

1. The total score can better reflect the severity of the disease. According to the data of the national scale collaboration group, its boundary

Values: more than 29 points may be serious anxiety; More than 21 points must have obvious anxiety; There must be more than 14 points

Anxiety; More than 7 points may cause anxiety; There are no anxiety symptoms when the score is less than 7; Generally, the boundary is 14 points.

2. HAMA is divided into two categories of factors:

Somatic anxiety consists of 7 items: 7, 8, 9, 10, 11, 12 and 13.

Psychic anxiety consists of items 1 to 6 and 14.

Factor score is the sum of the scores of the items that make up the factor/the number of items that make up the factor.
